# Prophylactic central neck dissection for cN0 papillary thyroid carcinoma: is there any difference between western countries and China? A systematic review and meta-analysis

**DOI:** 10.3389/fendo.2023.1176512

**Published:** 2023-07-27

**Authors:** Jiaxin Yang, Yuling Han, Yu Min, Cheng Chen, Jialin Chen, Ke Xiang, Jiahu Liao, Yang Feng, Daixing Hu, Guobing Yin

**Affiliations:** ^1^ Department of Breast and Thyroid Surgery, The Second Affiliated Hospital of Chongqing Medical University, Chongqing, China; ^2^ Department of Biotherapy and National Clinical Research Center for Geriatrics, Cancer Center, West China Hospital, Sichuan University, Chengdu, China

**Keywords:** papillary thyroid carcinoma (PTC), prophylactic central neck dissection (pCND), cN0, meta-analysis, effectiveness

## Abstract

**Background:**

Recommendations for the performance of prophylactic central neck dissection (pCND) in patients with clinically node-uninvolved (cN0) papillary thyroid carcinoma (PTC) are not the same. This meta-analysis set out to compare the effectiveness of pCND with total thyroidectomy (TT) in different countries and regions, mainly between western countries and China.

**Methods:**

The electronic databases PubMed, EMBASE, and Cochrane Library were searched for studies published until August 2022. The incidence rate of cervical lymph node metastases (LNMs), locoregional recurrences (LRRs), and postoperative complications were pooled by a random-effects model. Subgroup analyses based on different countries and regions were performed.

**Results:**

Eighteen studies involving 5,346 patients were analyzed. In the subgroup of western countries, patients undergoing pCND with TT had a significantly lower LRR rate [69/1,804, 3.82% vs. 139/2,541, 5.47%; odds ratio (OR) = 0.56; 95% CI 0.37–0.85] and a higher rate of temporary hypoparathyroidism (HPT) (316/1,279, 24.71% vs. 194/1,467, 13.22%; OR = 2.23; 95% CI 1.61–3.08) than that of the TT alone group, while no statistically significant difference was found in the rate of permanent HPT and temporary and permanent recurrent laryngeal nerve (RLN) injury. In the Chinese subgroup, the pCND with TT group had a significantly higher incidence rate of both temporary HPT (87/374, 23.26% vs. 36/324, 11.11%; OR = 2.24; 95% CI 1.32–3.81) and permanent HPT (21/374, 5.61% vs. 4/324, 1.23%; OR = 3.58; 95% CI = 1.24–10.37) than that of the TT alone group, while no significant difference was detected in the rate of LRR and temporary and permanent RLN injury.

**Conclusion:**

Compared with the TT alone for cN0 PTC patients, pCND with TT had a significantly lower LRR rate while having a higher temporary HPT rate in Europe, America, and Australia; however, it showed no significant difference in decreasing LRR rate while having a significantly raised rate of temporary and permanent HPT in China. More population-based results are required to advocate precision medicine in PTC.

**Systematic review registration:**

https://www.crd.york.ac.uk/PROSPERO/, identifier CRD42022358546.

## Introduction

Thyroid carcinoma (TC), the most common endocrine malignancy, has been diagnosed worldwide with increasing frequency in the last decades, especially among women (1–3). Most TCs are diagnosed when they are in a small volume or a diameter less than 1 cm by wild utilization of ultrasound and technical advances (4). The standard treatment regimens of TC include thyroid operation, adjuvant radioiodine (RAI) treatment, and thyroid-stimulating hormone (TSH) inhibition therapy (5). As the most common type of differentiated thyroid carcinoma (DTC), papillary thyroid carcinoma (PTC) exhibits an average incidence of 60% of cervical lymph node metastases (LNMs), which most commonly occur in the central neck compartment and are proven to be independent risk factors for locoregional recurrence (LRR) (6–9). Thereinto, up to 80% of metastases present with microscopic lymph node (7), resulting in a high LRR rate and affecting postoperative quality of life in up to 30% of patients with clinical node-negative (cN0) PTC (10, 11).

There is no controversy about the operation of therapeutic central neck dissection (CND) when clinical LNMs exist in the central neck compartment (12), while prophylactic CND (pCND) in cN0 PTC still remains controversial (13, 14). Recommendations of guidelines in different countries and regions are not the same. The 2015 American Thyroid Association (ATA) Guidelines stated that pCND (ipsilateral or bilateral) should be considered in patients with cN0 PTC who have advanced primary tumors (T3 or T4), clinically involved lateral neck nodes (cN1b), or if the information will be used to plan further steps in therapy (15). The 2019 European Society for Medical Oncology (ESMO) Clinical Practice Guidelines recommended pCND with total thyroidectomy (TT) to cT1-2N0 (Tumor less than 4 cm in greatest dimension, limited to the thyroid, and node-negative in clinical) DTC with childhood radiation exposure, family history, aggressive cytological features, multifocality and suspected extrathyroidal extension, and to more invasive tumors (T3 or T4) (16). The latest version of the NCCN Clinical Practice Guidelines in Oncology (NCCN Guidelines, Thyroid Carcinoma) did not recommend pCND if the cervical lymph nodes are negative in clinical (17). The 2016 Chinese guidelines suggested that primary tumor surgery with pCND is recommended with risk assessment before and during the operation, such as age, tumor diameter, and thyroid capsule invasion (18).

We suspect if there exists any biological difference of PTC among different ethnic groups in different countries and regions, manifesting as dissimilarities in terms of LNM, recurrences, postoperative complications, and survival rate, further influencing the formulation and revisal of guidelines. Therefore, this current meta-analysis was performed to compare the efficacy of pCND with TT for cN0 PTC in different countries and regions, mainly between western countries and China. We also compare the incidence rate of LRR, recurrent laryngeal nerve (RLN) injury, and hypoparathyroidism (HPT) between the pCND with TT group and the TT alone group in two different regions.

## Methods

### Protocol and guidance

This systematic review and meta-analysis followed the outline suggested by Preferred Reporting Items for Systematic Reviews and Meta-Analysis (PRISMA) (19). The registration number was CRD42022358546 on PROSPERO. Two authors independently searched the literature, extracted the data, and evaluated the study quality. In the process, controversial issues or conflicts were discussed, and disagreements would be solved by a third author.

### Search strategy

The PubMed, EMBASE, and Cochrane Library electronic databases were searched for relevant literature from inception to August 2022. The Medical Subject Headings terms were as follows: 1) Thyroid Cancer, Papillary OR Papillary Thyroid Carcinoma, 2) Neck Dissection OR prophylactic central neck dissection. Two terms were combined by “and.” The references of previous studies and identified trials were manually backtracked to broaden the search for related literature.

### Inclusion and exclusion criteria

The inclusion criteria were as follows: 1) study population should be PTC patients without LNM according to preoperative imaging and intraoperative inspection; 2) studies contained pCND with TT and TT alone groups; and 3) the incidence of central LNM, LRR, or complications could be available or calculated. Studies were restricted to those published in English.

Studies were excluded because of at least one of the following: 1) patients with clinically involved lymph nodes; 2) patients undergoing thyroidectomy previously; 3) patients undergoing other thyroid surgery besides pCND and TT; 4) publication region was not in China, Europe, America, or Australia; or 5) the full text was not available online.

### Quality assessment

For retrospective and prospective studies, the Newcastle-Ottawa Scale (NOS) (20) was adopted to evaluate the quality, which was based on three aspects, including selection, comparability, and exposure outcome condition. A score of 7–9 was described as high quality. Likewise, the quality of randomized controlled trials (RCTs) was estimated by the Jadad scoring system (21), which consisted of three parts, including randomization, blinding, and the description of withdrawals and dropouts. A score of 3–5 was regarded as high quality.

### Data extraction

Detailed characteristics of enrolled articles were extracted as follows: the first author, publication year, publication country, study design, study period, and follow-up duration. Baseline and clinicopathological data were acquired as follows: number of patients who underwent pCND with TT or TT alone, the lost to follow-up, age, sex, tumor size, extrathyroidal extension, multifocality, number of T3/4 patients, incidence of central LNM, and number of patients performing postoperative RAI treatment. The number of patients having LRR or postoperative complications (temporary and permanent RLN injury, temporary and permanent HPT) was obtained as the outcome data.

### Statistical analysis

Heterogeneity was assessed by I^2^ statistics and Cochran’s Q-statistic test with a p value. I^2^ > 50% and p < 0.1 would be deemed as significantly heterogeneous. Publication bias was assessed by Begg’s and Egger’s tests, and p < 0.1 was defined as a statistically significant publication bias.

Odds ratio (OR) and its associated 95% confidence intervals (CIs) were adopted to appraise dichotomous outcomes, and p < 0.05 was regarded as statistically significant. Combined with the result of heterogeneity analysis and study type, the Mantel–Haenszel random-effects model was exerted in this meta-analysis. Additionally, subgroup analyses were performed to estimate if there were differences in LRR and complications caused by the difference of ethnic groups and regions.

STATA software, version 14.0 (StataCorp LP, College Station, TX, USA) was exploited to statistical analyses.

## Results

### Study selection

**Figure 1:**
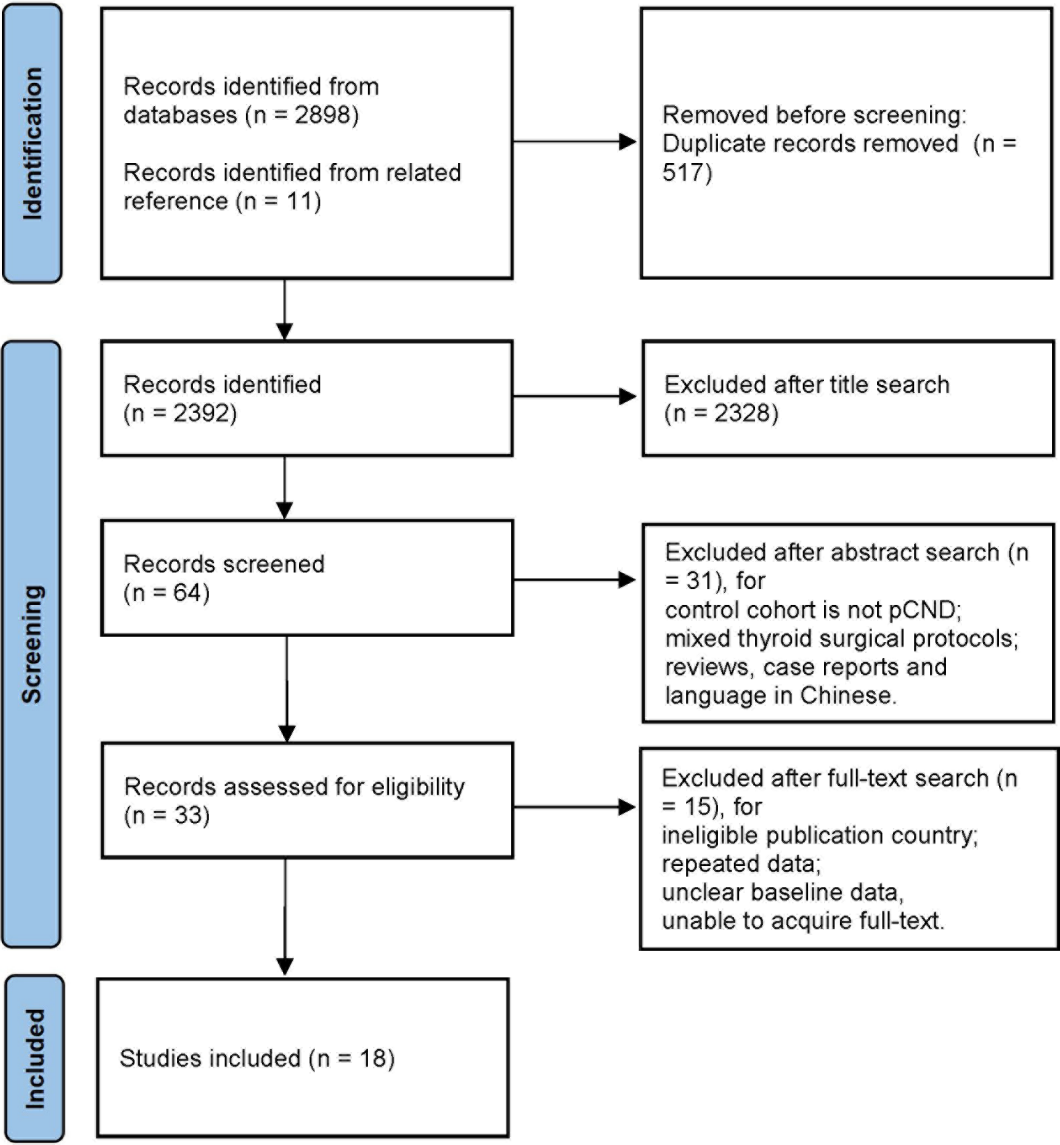
Flowchart of studies included with reasons.

A total of 2,909 studies were identified from three databases, among which 1,023 were from PubMed, 1,816 were from EMBASE, and 72 were from Cochrane Library databases. Eighteen studies were ultimately included, of which 14 were from western countries and four were from China (**Figure 1**).

### Study characteristics

All of the 18 included studies were published between 2010 and 2020, and there were 15 retrospective cohort studies (9, 12, 22–34), two RCTs (35, 36), and only one prospective cohort study (37). All studies were published in English language, and nine studies were performed in Europe, four in North America, four in China, and one multicenter study in Australia, America, and England.

A total of 5,346 patients with cN0 PTC underwent TT, of whom 2,341 were in the pCND with TT group and 3,005 in the TT alone group. Three studies (12, 23, 37) contained subgroups of unilateral pCND and bilateral pCND. The pCND with TT group in three studies (9, 22, 33) was mixed with unilateral and bilateral dissection. Nine studies (24–26, 28–32, 36) defined bilateral pCND as the lymph node dissection range. Three studies (27, 34, 35) defined the pCND with TT group as receiving only ipsilateral pCND. Two studies (12, 24) defined HPT and nerve palsy as permanent if they could not recover in 12 months after initial surgery, while other studies defined HPT and nerve palsy as permanent if they could not recover in 6 months after initial surgery.

Women accounted for most of the included patients. The mean tumor size ranged from 5.0 to 27.0 mm. The mean age at diagnosis was between 40.0 and 57.0 years old. The proportion of T3/4 PTC was not more than half. The proportion of PTC with multifocality and extrathyroidal invasion varied slightly because of the diverse inclusion criteria across studies (**Table 1**).

**Table 1:** Characteristics of the included studies.

Study	Country	Design	NOS score	Study period	Sample Capacity	Follow up (mean, month)	Age (mean, year)	Sex (M/F)	Tumor size(mean, mm)	T3/4, n(%)	Multifocali-ty, n(%)	Extrathyroidal extension, n(%)
Hughes et al., 2010 (25)	America	Retro	8	2002 -2009	TT+pCND:78	19.1^†^	46.8^†^	17/61	19.0^†^	NA	26 (33.3)	32 (41.0)
					TT:65	27.5^†^	41.2^†^	16/49	20.0^†^	NA	22 (33.8)	11 (17.0)
Moo et al., 2010 (28)	America	Retro	8	1999.07-2009.03	TT+pCND:45	37.2	45.7	10/35	14.2	NA	25 (55.6)	11 (24.4)
					TT:36	37.2	49.2	4/32	20.4	NA	23 (36.9)	13 (36.1)
Popadich et al., 2011 (38)	Australia England America	Retro	8	1995.01-2009.12	TT+pCND:259	32.0	44.0	52/205	22.7	80 (30.9)	125 (48.3)	72 (27.8)
					TT:347	50.0	48.0	81/266	22.3	96 (27.7)	148 (42.7)	84(24.2)
Lang et al., 2012 (27)	China	Retro	8	2004 -2010	TT+pCND:82	25.5^†^	52.0^†^	18/64	15.0^†^	35 (42.7)	30 (36.6)	22 (26.8)
					TT:103	27.1^†^	50.0^†^	22/81	10.0^†^	18 (17.5)	28 (27.2)	15 (14.6)
Raffaelli et al., 2012 (37)	Italy	Pro	8	2008.03-2010.10	TT+pCND:124	25.0	42.7	24/100	16.1	32 (25.8)	64 (51.6)	NA
					TT:62	25.5	43.2	13/49	15.3	15 (24.2)	29 (46.8)	NA
Barczyński et al., 2013 (24)	Poland	Retro	7	1993 -2002	TT+pCND:358	126.4	52.7	75/283	NA	47 (13.1)	135 (37.7)	NA
					TT:282	128.8	52.7	60/222	NA	36 (12.8)	99 (35.1)	NA
Hartl et al., 2013 (29)	France	Retro	8	1995 -2010	TT+pCND:155	82.8	46.0	39/116	NA	37 (23.8)	78 (50.3)	31 (20.0)
					TT:91	74.4	47.0	14/77	NA	18 (19.8)	39 (42.8)	14 (15.4)
Calò et al., 2014 (34)	Italy	Retro	8	2002.09-2010.10	TT+pCND:65	100.0	46.36	12/53	17.2	NA	20 (30.8)	27 (41.5)
					TT:220	100.0	53.07	48/172	16.1	NA	60 (27.3)	46 (20.9)
Kwan et al., 2015 (31)	China	Retro	8	1995 -2011	TT+pCND:54	59.0^†^	51.0	16/38	NA	11 (18.6)	NA	NA
					TT:51	48.0^†^	50.0	7/44	NA	3 (5.9)	NA	NA
Zhang et al., 2015 (9)	China	Retro	8	2008.01-2009.12	TT+pCND:134	61.0	48.0	26/108	7.0	NA	NA	4 (3.0)
					TT:108	66.0	45.0	27/81	5.0	NA	NA	2 (1.9)
Viola et al., 2015 (36)	Italy	RCT	4^*^	2008.01-2010.04	TT+pCND:93	60.0	45.7	25/68	16.0	44 (47.3)	39 (41.9)	44 (47.3)
					TT:88	60.0	43.5	21/67	16.0	44 (50.0)	44 (50.0)	42 (47.7)
Said et al., 2016 (26)	America	Retro	8	1996.01-2008.10	TT+pCND:34	94.8	40.0	10/24	27.0	NA	NA	NA
					TT:830	94.8	46.4	121/709	19.0	NA	NA	NA
Lin et al., 2017 (32)	China	Retro	8	2014.01-2015.08	TT+pCND:105	30.9^†^	NA	27/78	9.7^†^	30 (28.6)	54 (51.4)	33 (31.4)
					TT:62	27.6^†^	NA	12/50	11.7^†^	13 (21.0)	38 (61.3)	13 (21.0)
Calò et al., 2017 (12)	Italy	Retro	8	2008.01-2012.12	TT+pCND:85	71.9	42.02	15/70	15.1	NA	39 (45.9)	39(45.9)
					TT:173	56.3	47.8	40/133	17.8	NA	60 (34.7)	44(25.4)
Dobrinja et al., 2017 (22)	Italy	Retro	8	2000.01-2015.12	TT+pCND:74	37.0	53.0	12/62	13.0	28 (37.8)	NA	NA
					TT:112	76.0	57.0	29/83	11.0	25 (22.3)	NA	NA
Giordano et al., 2017 (23)	Italy	Retro	7	1984.01-2008.12	TT+pCND:405	113.0	49^‡^	135/475^‡^	NA	NA	NA	NA
					TT:205	113.0			NA	NA	NA	NA
Korkmaz et al., 2017 (30)	Turkey	Retro	7	2008.03-2015.07	TT+pCND:162	30.1	42.3	18/144	12.1	NA	69 (42.6)	25 (15.4)
					TT:140	30.7	49.6	20/120	13.2	NA	65 (46.4)	23 (16.4)
Sippel et al., 2020 (35)	America	RCT	4^*^	2014.06-2015.06	TT+pCND:29	12.0	50.1	7/22	19.1	4 (13.7)	14 (48.3)	8 (27.6)
					TT:30	12.0	46.1	7/23	24.5	7 (23.3)	13 (43.3)	5 (16.7)

Retro, retrospective study; Pro, prospective study; RCT, randomized controlled trial; pCND, prophylactic central neck dissection; TT, total thyroidectomy; NA, not available. ^*^evaluated by the Jadad scoring system. ^‡^characteristic of total enrolled patients. ^†^median.

Almost all of the studies provided good follow-up on patients, while two (31, 36) of those reported one and five patients lost to follow-up, respectively. The incidence of central LNM in the pCND with TT group of each article was counted. More patients in the pCND with TT group received postoperative RAI treatment compared with the TT alone group. Meanwhile, the postoperative RAI treatment showed obvious differences. In five studies (23, 29, 33, 35, 36), all patients were prescribed RAI adjuvant, while it was prescribed for patients with risk factors in the rest of the studies. The comparison of LRRs and postoperative complications between the two therapy groups was shown clearly in **Table 2**.

**Table 2:** Comparison of outcomes between the TT+pCND group and the TT alone group.

Study	Sample Capacity	Lost to follow-up	Incidence of central LNM, n (%)	Radioactive iodine treatment, n (%)	Locoregional recurrence, n(%)	Temporary RLN injury, n(%)	Permanent RLN injury, n(%)	Temporary HPT, n(%)	Permanent HPT, n(%)
Hughes et al., 2010 (25)	TT+pCND:78	0	48 (61.5)	72 (92.3)	4 (5.1)	NA	0	21 (26.9)	2 (2.6)
	TT:65	0		56 (86.1)	3 (4.6)	NA	2 (3.1)	5 (7.7)	0
Moo et al., 2010 (28)	TT+pCND:45	0	15 (33.3)	31 (68.9)	2 (4.4)	2 (4.4)	0	14 (31.1)	0
	TT:36	0		26 (72.2)	6 (16.7)	0	0	1 (2.8)	1 (2.8)
Popadich et al., 2011 (38)	TT+pCND:259	0	127 (49.0)	259 (100)	13 (5.0)	1 (0.4)	1 (0.4)	25 (9.7)	2 (0.8)
	TT:347	0		347 (100)	29 (8.4)	8 (2.3)	6 (1.7)	14 (4.1)	2 (0.6)
Lang et al., 2012 (27)	TT+pCND:82	0	45 (54.9)	62 (75.6)	3 (3.7)	3 (1.8)	1 (0.6)	15 (18.3)	2 (2.4)
	TT:103	0		63 (68.0)	3 (2.9)	0 (0.0)	1 (0.5)	9 (8.7)	1 (1.0)
Raffaelli et al., 2012 (37)	TT+pCND:124	0	44 (35.5)	90 (72.6)	1 (0.8)	1 (0.8)	1 (0.8)	53 (42.7)	1 (0.8)
	TT:62	0		37 (59.7)	0	0	0	11 (17.7)	0
Barczyński et al., 2013 (24)	TT+pCND:358	0	108 (30.2)	231 (64.5)	15 (4.2)	26 (7.3)	9 (2.5)	109 (30.4)	8 (2.2)
	TT:282	0		79 (28.0)	37 (13.1)	18 (6.4)	6 (2.1)	37 (13.1)	2 (0.7)
Hartl et al., 2013 (29)	TT+pCND:155	0	80 (51.6)	155 (100)	3 (1.9)	NA	2 (1.3)	NA	4 (2.6)
	TT:91	0		91 (100)	12 (13.2)	NA	2 (2.2)	NA	6 (6.6)
Calò et al., 2014 (34)	TT+pCND:65	0	20 (30.1)	65 (100)	2 (3.1)	2 (3.1)	NA	25 (38.5)	7 (10.8)
	TT:220	0		194 (88.2)	5 (2.3)	3 (1.4)	NA	55 (25.0)	10 (4.5)
Kwan et al., 2015 (31)	TT+pCND:54	1	36 (67.9)	NA	NA	2 (3.8)	0	11 (20.8)	2 (3.8)
	TT:51	0		NA	NA	5 (9.8)	1 (2.0)	9 (17.6)	1 (2.0)
Zhang et al., 2015 (9)	TT+pCND:134	0	51 (38.1)	NA	3 (2.2)	2 (1.5)	1 (0.7)	40 (29.9)	2 (1.5)
	TT:108	0		NA	9 (8.3)	1 (0.9)	1 (0.9)	10 (9.3)	0
Viola et al., 2015 (36)	TT+pCND:93	5	43 (46.2)	93 (100)	3 (3.2)	NA	4 (4.3)	NA	18 (19.4)
	TT:88	0		88 (100)	3 (3.4)	NA	7 (8.0)	NA	7 (8.0)
Said et al., 2016 (26)	TT+pCND:34	0	13 (38.2)	4 (11.8)	1 (2.9)	NA	NA	NA	NA
	TT:830	0		52 (6.3)	23 (2.8)	NA	NA	NA	NA
Lin et al., 2017 (32)	TT+pCND:105	0	59 (56.2)	87 (82.9)	2 (2.3)	NA	4 (3.8)	21 (20.0)	15 (14.2)
	TT:62	0		46 (74.2)	2 (4.3)	NA	0	8 (12.9)	2 (3.2)
Calò et al., 2017 (12)	TT+pCND:85	0	35 (41.2)	64 (75.3)	1 (1.2)	1 (1.2)	0	29 (34.1)	2 (2.4)
	TT:173	0		105 (60.7)	4 (2.3)	2 (1.2)	0	35 (20.2)	2 (1.2)
Dobrinja et al., 2017 (22)	TT+pCND:74	0	32 (43.2)	43 (58.1)	4 (5.4)	7 (9.5)	3 (4.0)	11 (14.9)	6 (8.1)
	TT:112	0		55 (49.1)	4 (3.6)	3 (2.7)	1 (0.9)	9 (8.0)	1 (0.9)
Giordano et al., 2017 (23)	TT+pCND:405	0	135 (33.3)	405 (100)	20 (4.9)	NA	3 (1.5)	NA	36 (22.7)
	TT:205	0		205 (100)	12 (5.9)	NA	1 (0.49)	NA	9 (4.59)
Korkmaz et al., 2017 (30)	TT+pCND:162	0	45 (27.8)	139 (85.8)	NA	NA	2 (1.2)	22 (13.6)	6 (3.7)
	TT:140	0		104 (74.3)	NA	NA	0	17 (12.1)	5 (3.6)
Sippel et al., 2020 (35)	TT+pCND:29	0	9 (31.0)	29 (100)	0	3 (10.3)	NA	7 (24.1)	NA
	TT:30	0		30 (100)	1 (3.3)	4 (13.3)	NA	10 (33.3)	NA

TT, total thyroidectomy; pCND, prophylactic central neck dissection; LNM, lymph node metastasis; RLN, recurrent laryngeal nerve; HPT, hypoparathyroidism; NA, not available.

### Study quality

The quality of the 16 retrospective and prospective studies was proven to be high with a score of 7 or 8. Two RCTs received scores of 4, which demonstrated a relatively high quality (**Table 1**).

### Incidence of central LNM

**Figure 2:**
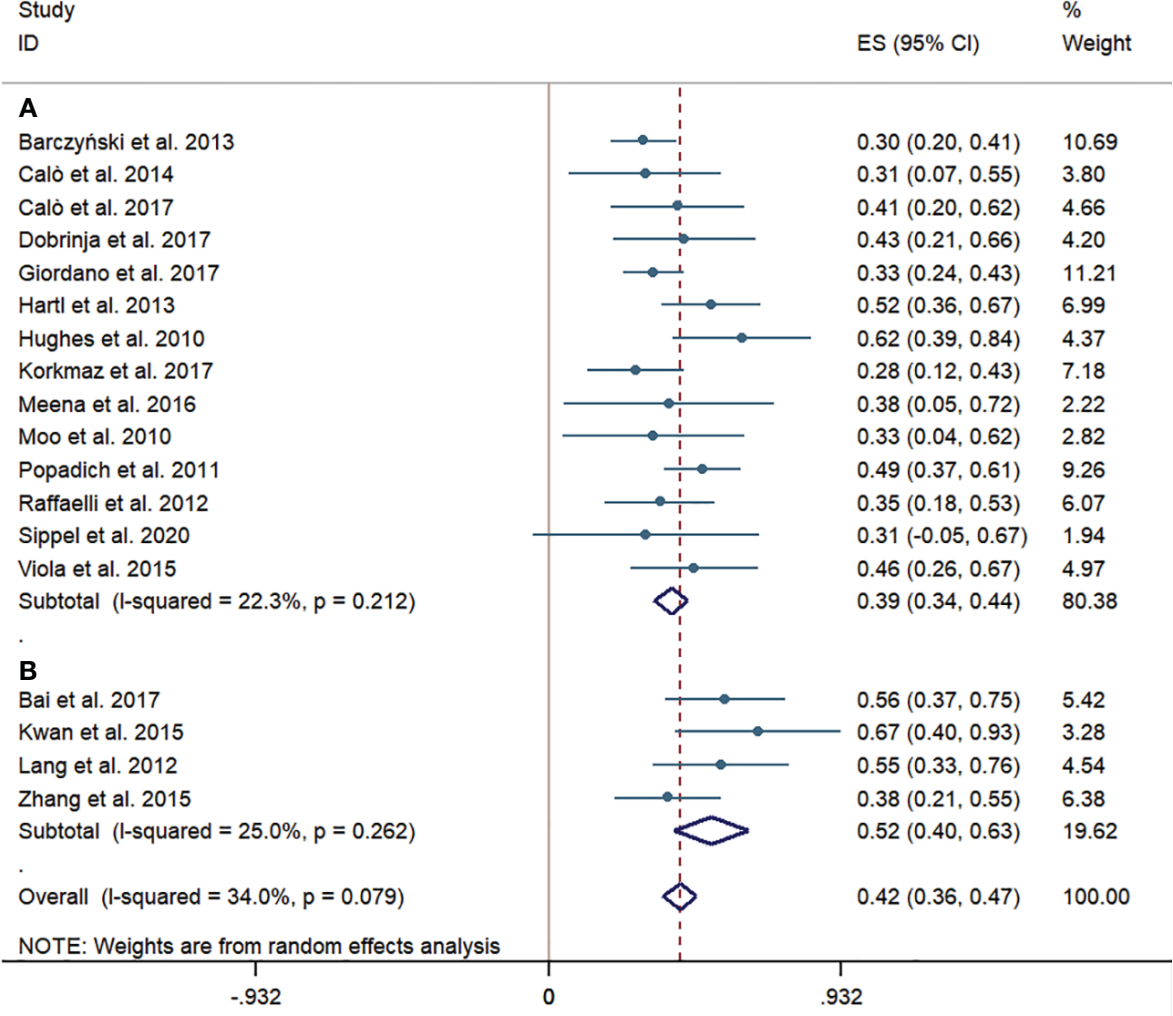
Forest plots for cervical lymph node metastases in the pCND with TT group. **(A)** Outcome of studies in western countries. **(B)** Outcome of studies in China. ES, effect size; CI, confidence interval; pCND, prophylactic central neck dissection; TT, total thyroidectomy.

Overall, the pooled central LNM rate of patients undergoing pCND with TT in 14 studies from western countries was 39% (95% CI 34%–44%). While the pooled central LNM rate of the pCND with TT group in four Chinese studies was 52% (95% CI 40%–63%). These subgroup analyses had no significant heterogeneity, with an I^2^ < 50% and p > 0.1 (**Figure 2**).

### Outcome of LRR

The LRRs were reported in 13 western-country studies and three Chinese studies. Acceptable criteria for recording recurrences by doctors were defined as positive blood test results with raised thyroglobulin (Tg), antithyroglobulin antibody (TgAb), or thyroid-stimulating hormone (TSH) concentration (22–26, 28, 29, 33–37); positive imaging findings on cervical ultrasound, computed tomography scan, or whole-body imaging (9, 22–28, 32, 33, 35–37); macroscopic disease at clinical examination (25, 27); and pathological evidence on excision or cytology (9, 12, 24, 26, 28, 33–35).

**Figure 3:**
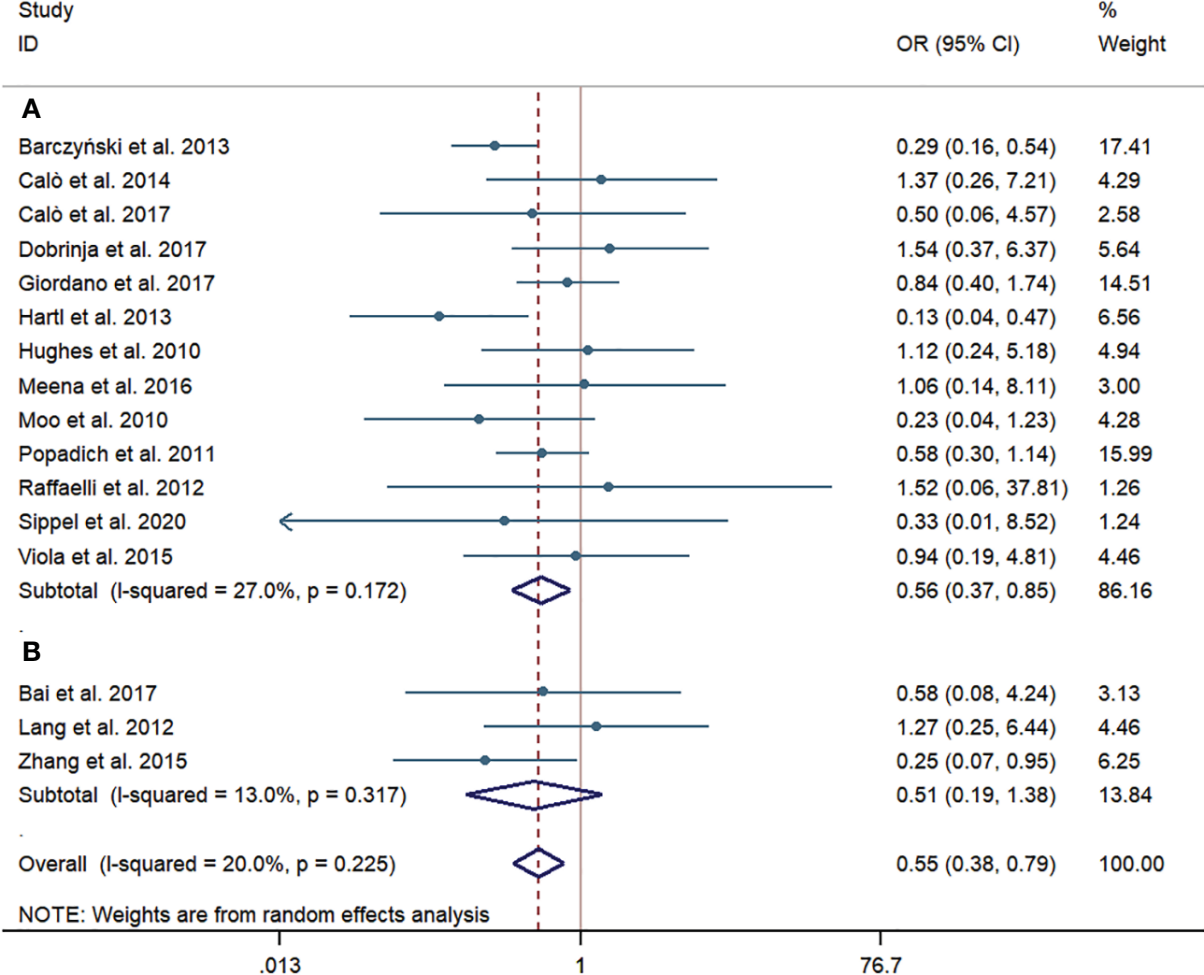
Forest plot for locoregional recurrences between the pCND with TT group and the TT alone group. **(A)** Subgroup analysis for studies in western countries. **(B)** Subgroup analysis for studies in China. OR, odds ratio; CI, confidence interval; pCND, prophylactic central neck dissection; TT, total thyroidectomy.

According to the subgroup of western countries, the pooled prevalence of LRR was significantly lower in the pCND with TT group than that in the TT alone group (69/1,804, 3.82% vs. 139/2,541, 5.47%; OR = 0.56; 95% CI 0.37–0.85). In the subgroup analysis of Chinese studies, the pooled prevalence of LRR was obviously lower in the pCND with TT group (8/321, 2.49% vs. 14/273, 5.13%); however, the difference was not statistically significant (OR = 0.51; 95% CI 0.19–1.38). There was no significant heterogeneity in the two subgroup analyses, and the publication bias confirmed by the Begg’s and Egger’s tests was not significant (Begg’s: p = 0.762, Egger’s: p = 0.329) (**Figure 3**).

### Temporary RLN injury

**Figure 4:**
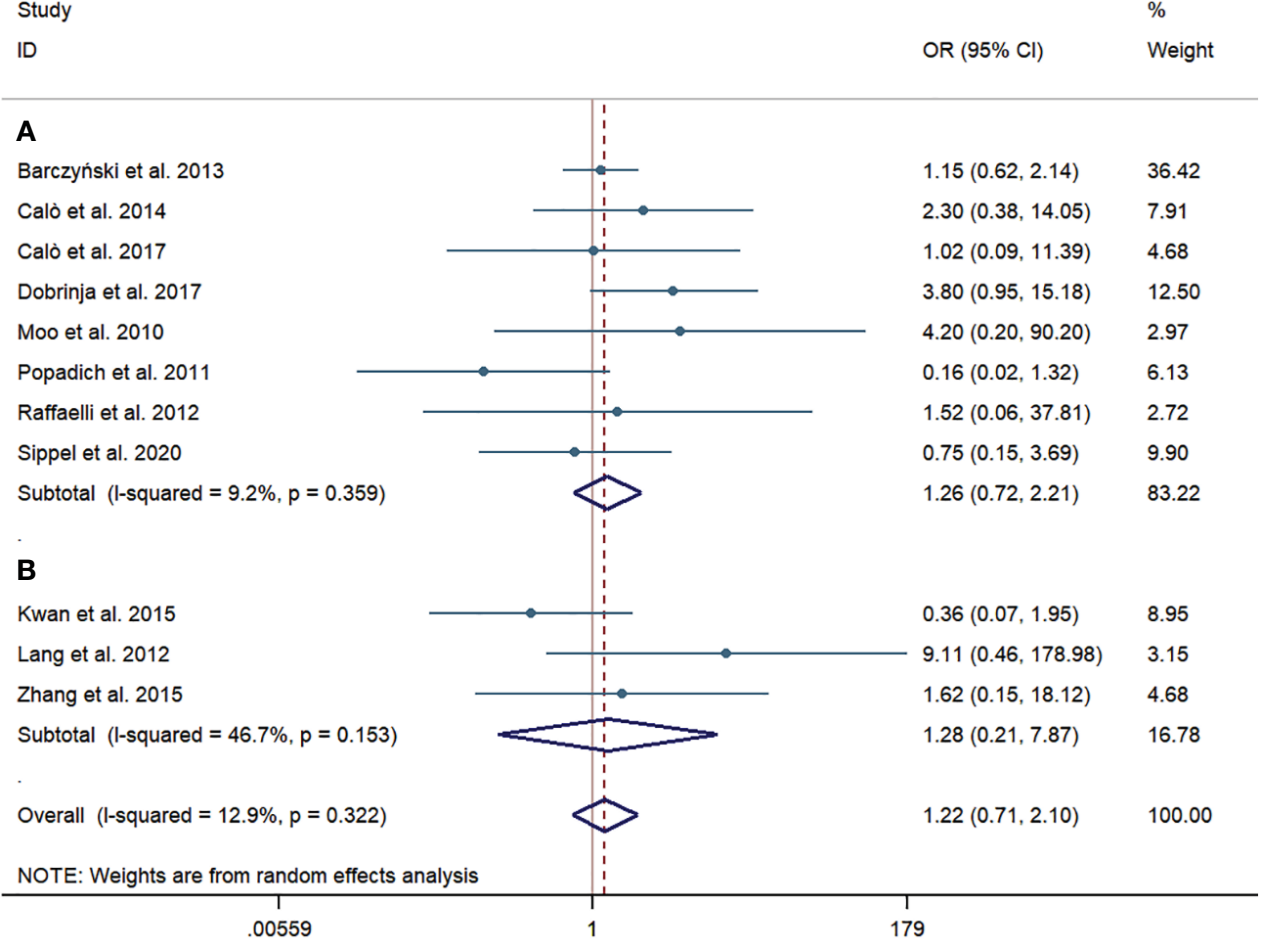
Forest plot for temporary recurrent laryngeal nerve injury between the pCND with TT group and the TT alone group. **(A)** Subgroup analysis for studies in western countries. **(B)** Subgroup analysis for studies in China. OR, odds ratio; CI, confidence interval; pCND, prophylactic central neck dissection; TT, total thyroidectomy.

Eight western-country studies and three Chinese studies assessed temporary RLN injury. The pCND with TT group did not show a significantly higher risk of temporary RLN injury than the TT alone group both in the subgroup of western-country research (43/1,039, 4.14% vs. 38/1,262, 3.01%; OR = 1.26; 95% CI 0.72–2.21) and in the subgroup of Chinese research (7/269, 2.60% vs. 6/262, 2.29%; OR = 1.28; 95% CI 0.21–7.87). No statistically significant heterogeneity was observed in subgroup analyses. The Egger’s and Begg’s publication bias tests showed that the bias statistic was not significant (Begg’s: p = 0.547, Egger’s: p = 0.735) (**Figure 4**).

### Permanent RLN injury

**Figure 5:**
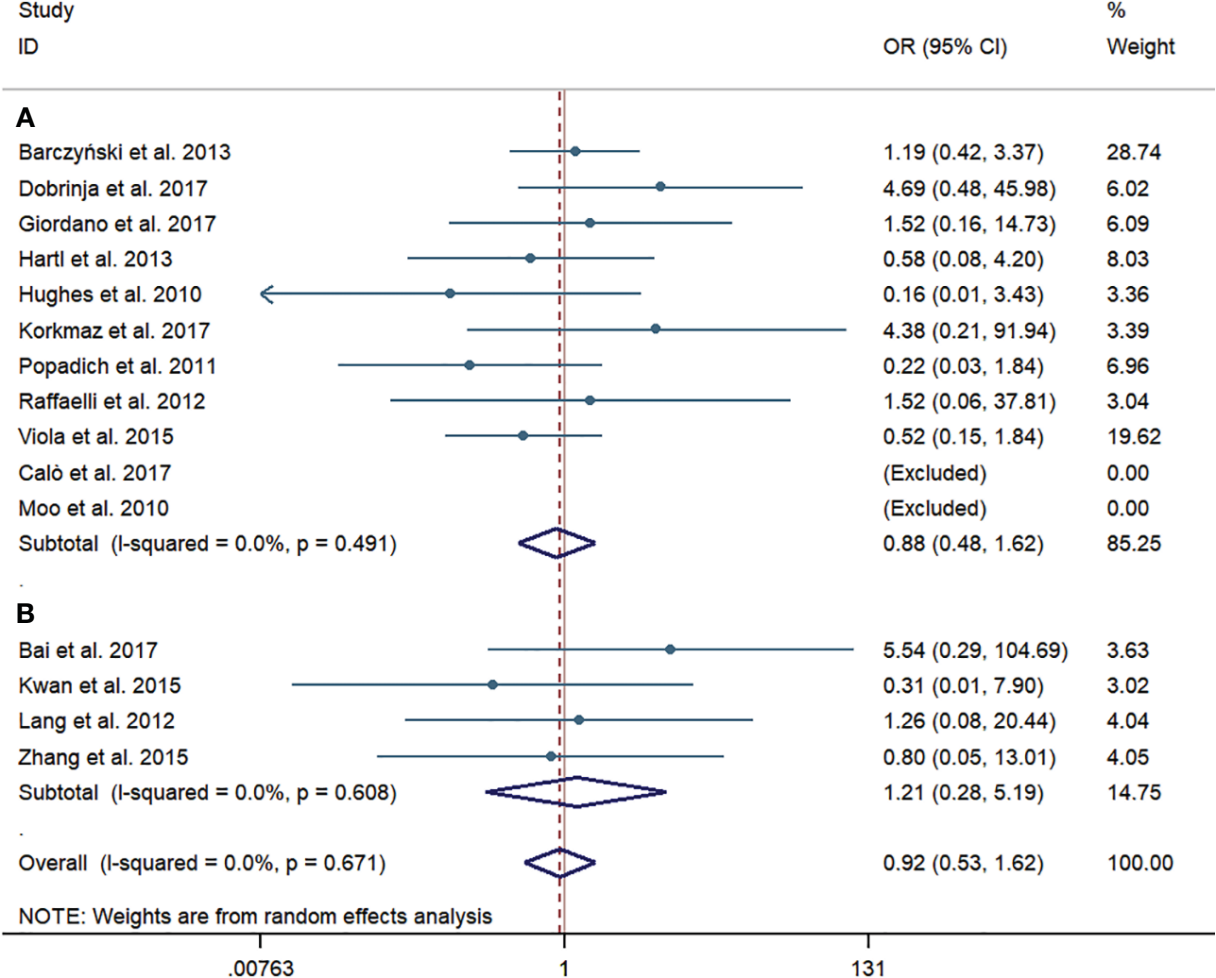
Forest plot for permanent recurrent laryngeal nerve injury between the pCND with TT group and the TT alone group. **(A)** Subgroup analysis for studies in western countries. **(B)** Subgroup analysis for studies in China. OR, odds ratio; CI, confidence interval; pCND, prophylactic central neck dissection; TT, total thyroidectomy.

Permanent RLN injury was investigated in 11 western-country articles and four Chinese articles. Like the results of temporary RLN injury, no significant difference was observed in the pooled incidence rate of permanent RLN injury between the pCND with TT and TT alone groups, according to the subgroup of western countries (25/1,838, 1.36% vs. 25/1,601, 1.56%; OR = 0.88; 95% CI 0.48–1.62) and the Chinese subgroup (6/374, 1.60% vs. 3/324, 0.93%; OR = 1.21; 95% CI 0.28–5.19). There was no evidence of heterogeneity in these analyses. No publication bias was detected by the Begg’s and Egger’s tests (Begg’s: p = 0.842, Egger’s: p = 0.897) (**Figure 5**).

### Temporary HPT

**Figure 6:**
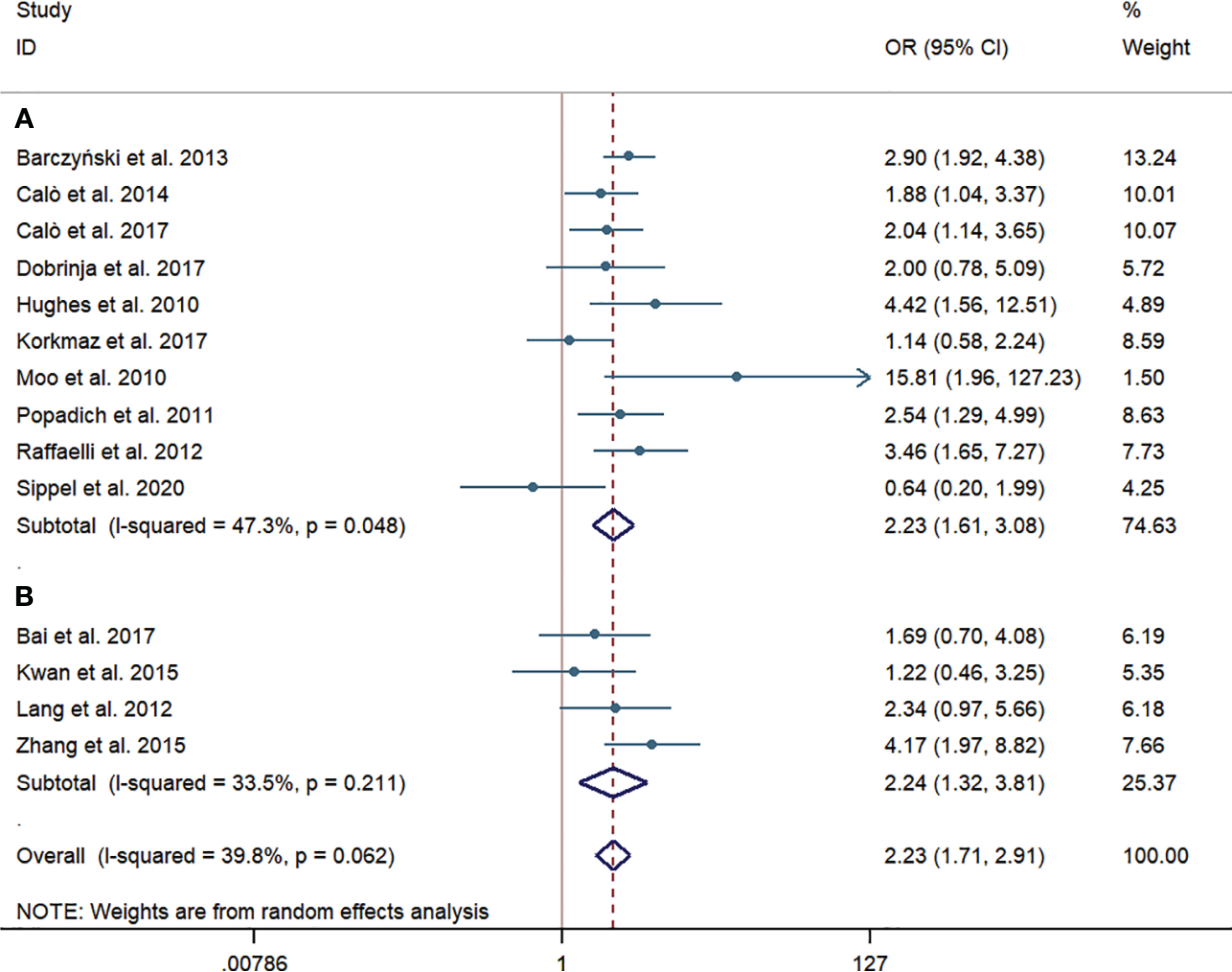
Forest plot for temporary hypoparathyroidism between the pCND with TT group and the TT alone group. **(A)** Subgroup analysis for studies in western countries. **(B)** Subgroup analysis for studies in China. OR, odds ratio; CI, confidence interval; pCND, prophylactic central neck dissection; TT, total thyroidectomy.

Ten western-country studies and four Chinese studies examined temporary HPT. For the pooled incidence rate of temporary HPT, the pCND with TT group demonstrated a significantly higher risk than the TT alone group, not only in the western country subgroup (316/1,279, 24.71% vs. 194/1,467, 13.22%; OR = 2.23; 95% CI 1.61–3.08) but also in the Chinese subgroup (87/374, 23.26% vs. 36/324, 11.11%; OR = 2.24; 95% CI 1.32-3.81). We encountered mild heterogeneity in the studies of western countries, with an I^2 = ^47.3% and p = 0.048; however, we found no significant heterogeneity in subgroup analyses of Chinese studies and no indication of publication bias among this body of articles (Begg’s test: p = 0.931, Egger’s test: p = 0.927) (**Figure 6**).

### Permanent HPT

**Figure 7:**
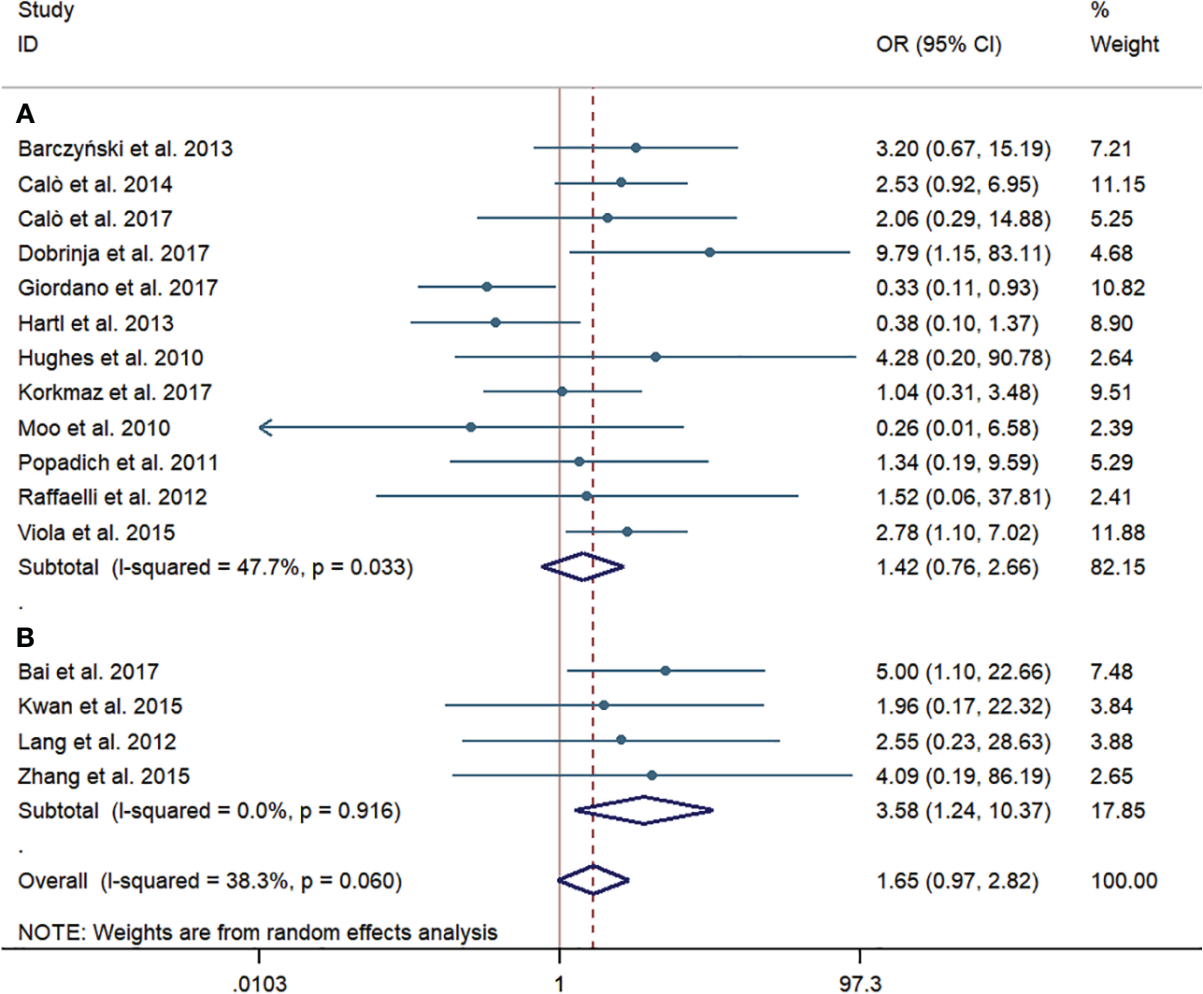
Forest plot for permanent hypoparathyroidism between the pCND with TT group and the TT alone group. **(A)** Subgroup analysis for studies in western countries. **(B)** Subgroup analysis for studies in China.

Twelve western-country studies and four Chinese studies evaluated permanent HPT. Similarly, the risk of permanent HPT was higher in the pCND with TT group than that in the TT alone group in the subgroup of western countries and China (62/1,903, 3.26% vs. 45/1,821, 2.47%; 21/374, 5.61% vs. 4/324, 1.23%). The difference between two groups failed to be statistically significant in the western country subgroup (OR = 1.42; 95% CI = 0.76–2.66), which had a slight heterogeneity, with I^2^ = 47.7% and p = 0.033. However, the difference was counted to be statistically significant in the Chinese subgroup (OR = 3.58; 95% CI = 1.24–10.37) with no statistically significant heterogeneity. Overall, no evidence of publication bias was found by the Begg’s (p = 0.687) and Egger’s tests (p = 0.412) (**Figure 7**).

## Discussion

To our knowledge, this meta-analysis to date firstly evaluated the efficiency of pCND with TT for patients with cN0 PTC in different countries and regions. The outcome of pCND reducing the risk of lymph node recurrence for cN0 PTC varies in different studies and still remains controversial. Both Zhao et al. (38) and Chen et al. (39) signified that the pCND with TT group had a significantly lower chance to experience LRR, whose meta-analyses contacted large samples and involved studies from all of the world, especially from Europe and America. These results are consistent with our finding in the subgroup of western countries. Inversely, Sanabria et al. (40) published a meta-analysis of five RCTs this year and failed to find a beneficial impact of pCND on locoregional or biochemical recurrence. The outcome of our analysis in the Chinese subgroup was similar to that new finding, although the initial surgery plan of the above study was thyroidectomy rather than TT only.

As we can see, there were just three Chinese articles reporting the difference of LRR rates between pCND with TT and TT (9, 27, 32) and the sample size was small, so it was hard to obtain a stable result on the LRR rate. Moreover, in the Chinese subgroup, the scope of pCND in three studies was defined as unilateral (27), bilateral (32), and a mixture of unilateral and bilateral (9). Although Chen et al. (39) deemed that bilateral or ipsilateral pCND seldom affects the LRR or complication rates, we still believe that the unstandardized operation scope of pCND might result in insufficient detection of potentially existing positive lymph nodes, especially the microscopic ones. Thus, the rates of LRR between two groups in the Chinese subgroup would not reach statistical significance, as it has been traditionally recognized that LRR is highly associated with cervical LNM (41). Moreover, inadequate information would be obtained to plan further steps in RAI treatment and TSH suppression treatment. In addition, inconsistent definitions of LRR might bias the results. These factors conjunctively may favor our findings that no significant difference was found in reducing the LRR rates of the pCND with TT and TT alone groups by subgroup analysis of Chinese articles.

In terms of postoperative complications, we found some similarities. In both western-country and Chinese subgroups, the difference of temporary and permanent RLN injury did not reach statistical significance between the pCND with TT and TT alone groups, while the pCND with TT group had a significantly higher rate of temporary HPT than that of the TT alone group. This finding was consistent with that of Henry et al. (42) who reported that approximately 14% of PTC patients presented temporary HPT and 4% of patients remained with permanent HPT after pCND with TT. With the development and application of advanced surgical instruments, such as harmonic scalpel, intraoperative nerve monitoring, and da Vinci robot, surgery will get more elaborate and lead to less nerve injury (43, 44). However, the wider dissection of the central neck compartment might cut off the blood supply to the parathyroids, extremely, the ipsilateral inferior gland (45). Moreover, during the pCND with TT, the occurrence of incidental parathyroidectomy or belated parathyroid gland transplantation would also cause postoperative temporary or even permanent HPT (46). These could probably explain what we found—that pCND with TT significantly increased the rate of permanent HPT in the Chinese subgroup.

According to the American and European guidelines, the attitude toward pCND for cN0 PTC patients in western countries is relatively conservative. According to our subgroup analyses of western-country literature, pCND has the characteristics of reducing recurrence and leading to relatively few severe complications, so why do not western guidelines consistently recommend it? After pCND with TT, LNMs were confirmed in 41% of PTCs, which indicated a moderate level of finding positive lymph nodes, as Rotstein (6) reckoned that the incidence of cervical LNMs ranges from 20% to 90% with an average of 60%. Moreover, it was hard to balance a 0.66% reduction in local recurrence with a 1.83% increase in temporary HPT. We are able to acquire from guidelines that patients with risk factors, who are recommended to undergo pCND along with TT, are also considered to receive postoperative RAI therapy after thyroidectomy without pCND. RAI therapy owns definite benefit in PTC patients with a high risk of recurrence or death (47); however, how much of pCND was attributable to the increased use of RAI therapy was not clear yet. Temporary HPT caused by the larger surgery extent could have a short-term influence on the quality of life, such as the numbness of the mouth and lips. Moreover, one latest literature states that incidence rates of thyroid cancer are now declining at a slight pace in America, owing to more conservative biopsy criteria and updated recommendations (1). Attitudes toward thyroid cancer in the western world seem to become increasingly conservative in terms of both diagnosis and treatment.

The consensus among Chinese experts and professors on pCND has been relatively radical from the past to now. As reported in our subgroup analyses of Chinese studies, the cervical LNM rate was 52%, which was higher than that in the western country subgroup (**Figure 2**). Considering that the unilateral and bilateral dissection was mixed in the Chinese subgroup, we speculated that this rate would be increased if all of the patients received a bilateral pCND. We further reckon that the biological characteristics of PTC in central LNM are more obvious among the population in China than in western countries, and whether population-based genetic detection could explain the problem. Thus, we are looking forward to further profound studies focusing on this issue. Xu et al. (48) found that up to 19.8% of patients experienced structural re-recurrence in about 5 years after reoperation, and Su et al. (49) and Zhao et al. (50) showed that approximately 6% of patients were diagnosed as permanent HPT after reoperation. Compared with reoperation, the recurrence and complication rates of pCND during initial TT were lower in the Chinese subgroup. Additionally, two Chinese studies indicated that 78%–96% of temporary HPT caused by initial TT would recover in a brief postoperative follow-up duration (51, 52). Therefore, pCND followed by TT is necessary to decrease the LRR rate and the possibility of the second operation.

In view of the excellent survival rates of patients with PTC (53), very few studies explored the overall or disease-free survival in this meta-analysis. Therefore, further high-quality work is required to investigate survival conditions between pCND with TT and TT alone, so that more convictive evidence will be used to compare the pros and cons of pCND. Moreover, it is profound to see other subgroup analyses of pCND for cN0 PTC based on factors, such as age at diagnosis, tumor diameter or volume, family history, and juvenile radiation history.

Certain limitations were also contained in this meta-analysis. First, retrospective studies accounted for the majority of the included research, and bias is inevitable. Second, the number of accessible Chinese articles in English databases is insufficient. Third, the different standards for pCND or RAI treatment probably introduced selection bias. Moreover, the surgical experience and skills of surgeons, even if in the same district, are rarely on the same level. Finally, as the included studies were from different countries and regions, the economic level (high-income countries were proven to have the highest incidence of thyroid cancer) (54), medical development (widespread use of medical apparatus and instruments improves the detection rates in various diseases), and living environment factors (for example, radiation exposure and iodine supply) might introduce potential bias.

## Conclusion

Compared with the TT alone for cN0 PTC patients, pCND with TT had significantly lower LRR rate while higher temporary HPT rate in Europe, America, and Australia. Alternatively, it showed no significant difference in decreasing LRR rate while significantly increased the incidence rate of temporary and permanent HPT in China. Further research is needed to estimate the value of pCND among different ethnic groups in different countries and regions. More population-based results are required to advocate precision medicine in thyroid carcinoma.

## Data availability statement

The original contributions presented in the study are included in the article/supplementary material. Further inquiries can be directed to the corresponding author.

## Author contributions

Conception and design: JY; Administrative support: GY; Provision of study materials: YH and CC; Collection and assembly of data: JC and JL; Data analysis and interpretation: JY and KX; Article writing: All authors; All authors contributed to the article and approved the submitted version.
